# Life as we don’t know it: plant life in the history and philosophy of science an introduction

**DOI:** 10.1007/s40656-026-00752-3

**Published:** 2026-07-13

**Authors:** Fabrizio Baldassarri

**Affiliations:** University Rome Tre, Rome, Italy

**Keywords:** Plant life, Plant behaviour, Plant intelligence, Democritus, Aristotle, Averroes, Bacon, La Brosse, Cavendish, Buffon, Darwin, Candolle

## Abstract

This introduction discusses the challenges plant life has represented in the history and philosophy of science, and outlines the topics dealt with by the contributors. Understanding plant life, behaviour, and nature reveals a crucial feature to deal with several corners of science and society, but also to deal with the environment.

Water, soil, and the earth’s green mantle of plants make up

the world that supports the animal life of the earth.

Although modern man seldom remembers the fact,

he could not exist without the plants…

(Rachel Carlson, *Silent Spring*, p. 51)

While playing a game, my daughter once asked if the mysterious word she had to uncover was a living being—and when I said no, she asked whether it was *at least* a plant. For a five-year-old, plants live in a way fundamentally different from animals and humans, in a twilight zone not entirely within the category of the living. Although she experiences that nature is somehow alive, grouping plant life together with animal life does not quite sit right with her—perhaps because her main experience of plants is tinged with potted plants that respond to human activity, and wither and die during the summer. Beyond the anecdote, the idea that plants are passive and decorative, lying in the background, rather than truly alive, frequently arises.^1^ Besides the ecological (and political) question—are plants merely decorative elements in our homes and cities?—which falls outside the scope of this special issue, a deeper philosophical question surfaces: how do we define the life of plants? Can it truly be said that they share in the same kind of life as animals and humans?^2^

In 2005 book *Life as we do not know it*, Peter Ward considers the possibilities of alien life, namely, extraterrestrial life, pushing us to rethink life beyond the human frame—and in this sense we should stop restricting the notion of life to *our life*. Although defining life on the Earth is no longer a challenge for scientists and philosophers, and it refers to matter that has biological processes, a vivid debate has recently concerned the life and behaviour of plants: do plants feel, perceive, desire, and think? In the past decades, plants have attracted a growing attention from scholarship—philosophical, historical and scientific—on plant behaviour—see the brief reconstructions in both Quentin Hiernaux’s and Paolo Pecere’s contributions to this special issue.^3^ Yet, while attention to plant behaviour and life has grown dramatically in the work of scholars, crucial gaps still persist—not only in the recent philosophical and scientific quarrels over plant behaviour and cognition, but also in the historical treatment of vegetation.

This special issue aims to re-centre plants in philosophical and scientific definition of life, while unearthing the roots of contemporary debates. Despite a general indifference in some histories of philosophy and science, plants have indeed enjoyed a more substantial presence throughout Western culture, not just as objects or commodities, but as a subject of philosophical inquiry and scientific investigation—the presence of plant philosophies throughout the centuries, for instance, is not uncommon, while this had been generally overlooked.^4^

Perhaps, one of the reasons of this historical neglect stem from the Aristotelian tradition, in which plant life has been defined as an *obscure* subject—this is the opening sentence of pseudo-Aristotle *De plantis*, somehow revived in 1973 Tompkins and Bird popular book entitled *The Secret Life of Plants*.^5^ Yet, this is not entirely accurate. If one looks into texts, such an obscurity is not utterly true, as scholars have usually investigated and paid close attention to the complexities of plant life much more than what is historically acknowledged—thus making plant life a less obscure topic than what historians have rapidly defined. As a result, plants appear provided with more activities than merely staying that is, *vegetating* in the background, therefore playing a more prominent role in nature.

The definition of plant life as *vegetation* [in Latin: *vegetare*] has profoundly shaped Western philosophy and science. Traditionally, scholars have identified plant life by means of the basic activities of living nature, namely, nutrition, growth and reproduction—which Aristotle conceived as activities principled by the vegetative soul, and thereby common to all living beings. Besides a reductive interpretation of vegetative activities as such, according to which *vegetation* is the beginning of life and the first step in the traditional scale of nature, an alternative interpretation has developed. Recently, historians and philosophers have read this definition in a more inclusive way, according to which the life of plants comprises the lives of all living beings, characterizing its foundation.^6^ This latter interpretation has made plants central to life itself. The idea of vegetation underpinning all life has led contemporary scientists and phytobiologists to describe plants as *constructors of the environment where we live*—producers of nature, life and living condition.

What results is clear: understanding *vegetation* is key to understand life tout court, while it remains a notion under construction.

Still, several problems remain unresolved as not all interpretations of the scale of nature have consisted in a hierarchical restriction of plant life to a smaller amount of life that stems from the Aristotelian interpretation. If, on the one hand, plants display fewer living activities and a simpler physiology, it is also true that they live longer than animals, and adapt better to the various habitats—in some cases, scholars have labelled these features as evidences of plant superiority, sometimes extending it to moral, social, and political aspects.^7^ Additionally, scholars have used plants as models to explain a few living activities such as animal and human nutrition and growth, following an analogical reasoning. This was a common enterprise since Hippocrates, the ancient Greek physician living in Cos. The analogy between animals and plants has had 2 main consequences: the first is that plants were conceived as simpler bodies than animals, making it easier to explain living activities such as generation and growth (see Marilena Panarelli’s contribution to this fascicle); the second is that scholars conceived plants as truly representative of specific segments of life, making them as models of animal life as such, which is *vegetal* before being animal, as it happened for William Harvey in the seventeenth century and George-Louis Leclerc de Buffon in the eighteenth century (see Stella Sandford’s article in this fascicle).^8^

Yet, while plant life has been mostly overlooked by historians—this is not an easy subject. Problems and questions surface now and then. A first problem occurs when *vegetatio* refers to a general life process and not specifically to plants. This separation appears crucial throughout the centuries, as it blurs the line between nature and living beings (see Baldassarri’s and Sandford’s contribution in this special issue), and complicates any firm definition of plant life—if *vegetatio* does not specify plants but life in general, any attempt to theorize vegetal life tends to fail.

The second problem concerns the amount of plant activities and behaviour. Since plants live longer than animals, according to Western philosophies this might be due to the fact that plants consume less heat than animals—plants do not move, for instance, and some of them shed their leaves in autumn and winter—or that their principles lie outside them—the heating principle is the Sun, while in animals this is the heart; and the earth prepares food for plants, which only have to attract nutrients with their roots, while animals and humans digest food in the stomach. Both the absence of motion and the absence of an internal source of heat and nutrition disclose interpretative challenges. Both plant psychology and physiology are simpler and less individuated than the animal one, and this might entail that some activities merely occur outside the plant body—thereby restricting its *life* in a physiological perspective.^9^

Nonetheless, this aspect reveals a deep interaction between plants and the environment, as plants adapt to the different climates, reacting to the Sun and heat and responding to autumn and cold. Scholars frequently recognized the importance of such interactions—pre-Socratic philosophers such as Democritus investigated what differences were connected to the environmental conditions, while endowing plants with so-called superior faculties, namely, sensation, imagination, and cognition. These two issues are not disconnected. The concept of the soul-world (*anima-mundi*) importantly shaped the definition of plant activities at large, which in the Platonic tradition (widespread during the Renaissance and seventeenth century) extended to sensation, motion, imagination, and thinking. Yet, providing plants with a dynamic and interactive life reveals the interconnections between vegetal bodies and the environment—as the former shape the latter.

A third problem thus emerges: while Aristotelian tradition restricts vegetal activities to the basic operations of life, namely, nutrition, growth, and generation, Platonic tradition (later thriving in Medieval time under the influence of Averroes, among others, and in the Renaissance) proceeded further, expanding plant life—making plants not only lasting longer than animals, but also larger when compared to them. For instance, the fact that insects, fungi, and parasites live in plants—in what is today called symbiotic unity: a plant is an ecosystem—reveals that the life of plants is more encompassing than the one of animals or humans. Moreover, plant life develops both within the earth and on the earth, connecting the underground with the heavens—the mineral world with the stars—in ways that are not only metaphorical. This case has philosophical and scientific significance, as scholars compared the formation of plants to crystals and minerals, thus connecting vegetal and mineral nature. A more relevant case concerns the formation of insects such as galls on the leaves of plants, which philosophers conceived to be caused by the same life principle animating vegetables, thereby underlying the idea of a unified nature in which all creatures live from the same principles.^10^ This transformed the philosophical picture of life and nature, not as a mere hierarchy, but as a network of mutual co-existence principled by the same system.

Focusing on plant behaviour, the formation of insects and parasites in plants led thinkers to speculate in what ways a plant (with a vegetative soul) could generate an animal (with a sensitive soul), thereby offering crucial content to discuss spontaneous generation. Indeed, scholars conceived that also plants should possess a proto-sensitive soul and a form of sentience, ranging from appetite and desire—the mere desire for food, an interpretation already present in Plato’s philosophy—to sensation, motion, and forms of cognition. Throughout the history of philosophy and science, debates about plant appetite and desire, sentience and cognition reveal the complexities of defining plant life and the puzzles of interpreting the latter through the lens of animal life, again following the plant-animal analogy.^11^

Ancient physician Galen of Pergamum offered an alternative perspective. Drawing from the physiological analogies between plants and the liver, he claimed that the foetus is a plant in the very beginning, thereby making the plant a model to investigate foetus life.^12^ This interpretation was revived in pre-modern times at large, when scholars confined plant life to a short segment of animality, the one in which immobility and the absence of desires and passions characterizes life, and surfaced again in the work of Indian physiologist, Jagdish Chandra Bose, for instance.^13^ Yet, within the same tradition, philosophers also conceived plants as inverted humans—plant roots are like animal brains—suggesting a powerful metaphor spanning through different interpretative lines, up to Charles Darwin (see Pecere in this special issue), and to contemporary definition of plant intelligence.

But plant life also thrived outside the analogy with animal and human lives—that is, besides such analogical reasoning. Some authors argued about the diversity of plant life and behaviour. Since Antiquity, and throughout the Middle Ages and the early modern time, a few scholars have followed this path, claiming that plants possess their own senses—sometimes more than five—and their own system of communication—as recently explored, for instance, by Sumana Roy in *How I Became a Tree*: “I had, in frustration with industrial noise and human verbosity, mistaken trees as silent creatures […] my new realization—that trees shared a natural sound with people.” Yet, as Roy suggests, this is “the sound of resistance.”^14^

In this sense, scholars have argued that plants possess their own system of life, warning about the challenges of reducing it to the human and animal terms alone. This interpretation has developed from the physiological and philosophical interpretations to the effort of defining a different order in nature—a resisting and fighting one, which challenges anthropocentrism, rationalism, and modern mechanism, but also human societies and political organization. This order of nature is thus distant from the zoo-centric scale of beings developing as a ladder, but reveals an (eco-)system developing as a shrub of interconnected branches.^15^

As a result, while outlining the life and behaviour of plants, a different picture arises. The scholarly involvement with plants has offered an alternative way of imagining life, nature, and sociality. Observing the ways plants live together, building ecosystems and encompassing different forms of life, pre-modern and modern scholars have seen in plants the possibility of a model for alternative social systems, ultimately developing an ecological approach to social life drawing from plants.^16^ Distancing from the traditional criteria of Western civilization, the life of plants has become a model to subvert hierarchies, ultimately revealing a possible alternative to a technological world—thus outlining a crucial help to deal with contemporary challenges, such as the climate change.^17^

The contributions to this special issue do not extend this far, but concentrate on the philosophical and scientific engagement with, and definition of, plant life spanning from pre-Socratic philosophy to the contemporary involvement with plant behaviour, somehow highlighting how new generated questions intersect with traditional claims. Authors discuss two key aspects: The first regards plant generation and life expectancy, while the second concerns plant sentience and intelligence. These features proceed well-beyond plant life and pave the way to a different understanding of nature.

Ancient interpretations about plant generation and life have influenced Western thought at large, ranging from pre-Socratic interpretations surfacing in Anaxagoras and Democritus, and later Platonic interpretation, who attributed plants with an enlarged set of living faculties including sensation, communication, and cognition, and the Aristotelian restriction of plant life to the vegetative soul. Aristotle’s attempt to provide a metaphysical framework to biology importantly emerges, while difficulties in providing a specific definition for plant life reveals difficulties in relating vegetation with plant life.^18^ Later in Galen, the Aristotelian *scientia de anima* is reduced to the physiology of heat and humid, to the comparison of animal and plant anatomies and living functions, and to the environmental issues that have surfaced in Theophrastus’s work. In the opening article, Marilena Panarelli deals with the presence of the ancient tenet that the foetus in its first stages is a plant, focusing on a Medieval commentary on Hippocrates’ *De natura foetus* [On the nature of foetus] by Dino del Garbo (1280–1327). While expanding the analogies between plant and animal generation, del Garbo explored seed anatomy, the various stages of plant growth, and the sexual differentiation between plants from a physiological perspective.^19^ He offered a crucial interpretation about the mechanism and functional role of seed leaves (later called, Cotyledons, an important part of plant physiology—for instance surfacing in Marcello Malpighi’s *Anatome plantarum* [1675–1679],) combining this anatomical study with Aristotelian philosophical principles of life—i.e., inner heat and natural moisture. In studying the seed, del Garbo also succeeded in differentiating between perfect and imperfect generation—explant or spontaneous generation. This sets beyond the plant-animal analogy, as the re-generative capacity of vegetation, whose severed limbs may continue absorb nutrients and live when explanted, outlines the metaphysical and physiological richness of plant life.

Within this line, Stella Sandford explores the implications of studying plant reproduction in the work of eighteenth-century naturalists George-Louis Leclerc de Buffon. Despite the huge contextual and scientific differences, Buffon’s investigation into plant generation remains influenced by Aristotelian concepts—stretching the animal-plant analogy to the claim that plant generation is a model for animal generation. Indeed, he aims to fill a crucial lacuna in the studies of plant life, bridging the gap between vegetables (*les végétaux*) and vegetation (*vegetation*) that affected Western philosophy and plant studies since Aristotelianism,^20^ while at the same time Buffon explored the different kind of plant generation, challenging preformationist theories, and discussed plant sexuality. Buffon asserted the centrality and superiority of plant life, rejecting zoo-centric interpretations, and also subsuming sexes under a vegetal model. This opens up to an alternative understanding of life, further explored throughout the centuries and not disconnected from the notion of vegetative soul and its faculties.^21^

Separating from the vegetative soul and its basic functions, namely, nutrition, growth and generation, the next 3 articles deal with the study of plant sentience and intelligence in different contexts. Fabrizio Baldassarri opens this section with a study of seventeenth-century attempts to attribute sentience, appetites, and cognitions to plants, focusing on authors such as Francis Bacon, Guy de La Brosse, and Margaret Cavendish. Challenging Cartesian mechanism, these authors develop an alternative interpretation of nature, drawing from Renaissance Italian naturalisms, according to which plant life is a key element to conceive nature and life under a new light—confirming the panpsychist notion that life is widespread through nature. Indeed, while expanding plant life and behaviour, scholars challenged Aristotelian hierarchical scale-of-beings, and the difficulties in relating vegetable life [*vegetatio*] with vegetables [*plants*], ultimately opposing zoo-centric and anthropomorphic beliefs. In these cases, plants acquired a more central position, outlining a different interpretation of nature that anticipates ecological philosophy.

Quentin Hiernaux, then, discusses the work of nineteenth-century plant biologist Augustin-Pyramus de Candolle, who attributed excitability, irritability and sentience to plants. On the one hand, de Candolle maintained a basic Aristotelian interpretation of nature, as the division between physical and vital reflects the division between plants—a simpler and more suitable model—and animals—whose instincts, feelings and consciousness obscure the basic biological processes. On the other hand, his light experiments provided him with a different understanding and were later important in the work of Charles Darwin on the movement of plants and in Paco Calvo and Monica Gagliano’s studies of plant sensation. De Candolle proposed three levels of biological organisation, providing a deeper explanation of plant behaviour and avoiding a strict analogy with animals—this latter remains a problematic feature throughout the time. His investigation into plant behaviour and life contributed to his work on plant taxonomy, and his understanding of evolution and phylogenetic classification, which proved important in nineteenth-century studies.

In the final contribution to this special issue, Paolo Pecere discusses the debates surrounding plant sentience and intelligence before and after the publication of Darwin’s 1880 *The Power of Movement in Plants*. While Darwin incorporated traditional notions such as irritability and panpsychism with his experimental investigation of plant life, and pursues the animal-plant analogy—his claim that the roots are like the animal brains is not only a common feature since Antiquity, but also a point of departure for contemporary plant neurobiology—significant differences from the tradition arise. A crucial shift concerns the rejection of metaphysical speculations and non-chemical studies of plant life, as Darwin grounded plant circumnutation on chemical processes that direct phototropism. This is a significant point of departure for contemporary philosophy and sciences of life, ultimately developing crucial views about cognitive ethology, behaviourism, ecology, but also treating plants as a community and not mere individuals—thus challenging anthropocentrism and supporting a more integrated, ecological view of life and nature.

If contemporary debate on plant behaviour provides a vital space to address today’s environmental crisis and related questions, this special issue aims to demonstrate how far these insights are rooted in a long tradition, spanning the centuries, from ancient plant studies to pre-modern and modern investigations on plants. Indeed, investigations into plant behaviour, sentience and intelligence, regeneration, growth, and nutrition, namely the topics treated by the contributions put together in this fascicle, have long shaped the scientific and philosophical conceptions of nature over the past 20 centuries—ultimately framing botanical practices in a more profound way than generally recognized.^22^ Yet, the articles collected here also reveal a deep relationship between plants and the environment, as well as plants and animals and humans. This interpretation distances from Heidegger’s failed encounter between humans and plants,^23^ and points toward the necessity for a unified conception of nature, counteracting Heidegger’s experience with what Monica Gagliano has defined an unexpected encounter between scientific experience and the indigenous wisdom of the plants. Such encounters suggest a more unified and respectful vision of nature, recognizing the complexity, vitality and intelligence of vegetation—ultimately shaping plant life as a crucial topic in the history and philosophy of science, and the environment.

## Footnotes

1 See Rix (2026) for a deeper investigation of plant activity.

2 Of course, these questions have an ecological-environmental perspective to be investigated in further research.

3 See also Calvo and Laurence (2023). Roy (2021). Gagliano (2018). Tassin (2018). Trewavas (2014). Chamovitz (2012).

4 While this is an important aspect, in this special issue contributors focus on the life of plants alone, avoiding to engage with this broader topic. More recently, philosophers have devoted important attention to this issue, see Marder (2013). Hiernaux and Timmermans (2018). Hiernaux (2021). See also Sandford (2022).

5 On this text, see Ferrini and Giglioni (2020). This idea surfaces in Wohlleben (2017), who clearly rejected this aspect.

6 This has been the reading of Mix (2018) and Baldassarri and Blank (2021).

7 See Brancher (2015) on this aspect. Giglioni (2023).

8 William Harvey introduced the idea of a *primordium vegetale* to explain the first moment of life, see Baldassarri (2024), pp. 101–105. Cf. Cunningham (2022).

9 See Hiernaux (2026).

10 This aspect intersects the notion of spontaneous generation, which gained attention in the seventeenth century, and reveals crucial in the definition of life.

11 Carvallo and Macé (2023); Carvallo and Macé (2024).

12 Vinkenstejin (2021); Pietrobelli (2025).

13 Bose (1908); Tandon (2019).

14 Roy (2021) p. 25.

15 Irigaray (2008).

16 Nealon (2016). Coccia (2024).

17 Gilles Deleuze and Félix Guattari have pointed out the ways rhizomes reveal the plant’s attempts to subvert hierarchies, see Thayer-Bacon (2017).

18 Falcon (2024).

19 On plant sexuality later in pre-modern times, see Sandford (2022).

20 Touwaide (2023).

21 Miller (2002).

22 As shown in this special issue, this interpretation challenges Michel Foucault’s claim that natural historians were not interested in defining the life of plants, but mainly busy in collecting specimens.

23 Marder and Roussel (2012).
